# The properties and mechanism of action of plant immunomodulators in regulation of immune response – A narrative review focusing on *Curcuma longa* L.*, Panax ginseng* C. A. Meyer and *Moringa oleifera* Lam

**DOI:** 10.1016/j.heliyon.2024.e28261

**Published:** 2024-03-21

**Authors:** Muggunna Balasubramaniam, Sarah Sapuan, Ilie Fadzilah Hashim, Nurul Izza Ismail, Amira Suriaty Yaakop, Nur Azzalia Kamaruzaman, Ana Masara Ahmad Mokhtar

**Affiliations:** a Small G protein Research Group, Bioprocess Technology Division, School of Industrial Technology, Universiti Sains Malaysia, 11800 Gelugor, Penang, Malaysia; b Department of Toxicology, Advanced Medical and Dental Institute, Universiti Sains Malaysia, 13200 Kepala Batas, Penang, Malaysia; c Department of Clinical Medicine, Advanced Medical and Dental Institute, Universiti Sains Malaysia, 13200 Kepala Batas, Penang, Malaysia; d School of Biological Sciences, Universiti Sains Malaysia, 11800 Gelugor, Penang, Malaysia; e National Poison Centre, Universiti Sains Malaysia, 11800 Gelugor, Penang, Malaysia; f Green Biopolymer Coating and Packaging Centre, School of Industrial Technology, Universiti Sains Malaysia, 11800 Gelugor, Penang, Malaysia

**Keywords:** Traditional plants, Immunomodulation, Immunosuppression, Immunostimulation, Phytochemicals

## Abstract

Herbal treatments have been utilized for millennia to cure a variety of ailments. There are over 20, 000 herbal remedies available to treat cancer and other disease in humans. In Ayurveda, traditional plants having revitalizing and nourishing characteristics are known as "Rasayanas." They have anti-inflammatory, anticancer, anti-microbicidal, antiviral, and immunomodulatory effects on the immune system. Immunomodulation is a mechanism through which the body stimulates, suppresses, or boosts the immune system to maintain homeostasis. Plant-derived immunomodulators are typically phytocompounds, including carbohydrates, phenolics, lipids, alkaloids, terpenoids, organosulfur, and nitrogen-containing chemicals. Immunomodulation activity of phytocompounds from traditional plants is primarily mediated through macrophage activation, phagocytosis stimulation, peritoneal macrophage stimulation, lymphoid cell stimulation, and suppression or enhancement of specific and non-specific cellular immune systems via numerous signalling pathways. Despite extensive research, the precise mechanism of immunomodulation of most traditional plants has not yet been fully elucidated, justifying the need for further experimentation. Therefore, this review describes the immunomodulatory agents from traditional plants such as *Curcuma longa* L., *Panax ginseng* C.A. Meyer, and *Moringa oleifera* Lam, further highlighting the common molecular targets and immunomodulatory mechanism involved in eradicating diseases.

## 1 Introduction

Immune system is a sophisticated defence network comprised of cells, tissues, and organs that operate in concert to protect the body against infectious agents such as microbes or viruses, thereby, maintaining immunity [1]. The word “immunity” is derived from a Latin word called *immunis*, meaning exempt [2]. Immunity is achieved when the immune system recognizes and tolerates substances that belong to the body or host (self) while rejecting non-self-substances (foreign particles or pathogens) [3]. The immune system establishes tolerance and achieves immunity with the aid of two immune subtypes, namely, the innate immunity and adaptive immunity [4]. Innate immunity is a non-specific immune response that includes physical and chemical barriers and cellular responses. It acts as a first line of defence by rapidly responding to the entry of foreign particles through mediators such as neutrophils, monocytes, dendritic cells and macrophages [5]. On the other hand, adaptive immunity normally involves the B and T lymphocytes and is characterized as a specific immune reaction that is stimulated by innate immune reaction [6]. Both innate and adaptive immunity work in concert to achieve tolerance and immunity. Malfunctions, imbalances or defects in either of these systems often lead to loss of tolerance and immunity, eventually compromising the immune system. Therefore, individuals with compromised immune system are at higher of risks of developing life-threatening chronic diseases such as allergies, autoimmune diseases, and cancer. Although several forms of treatment exists, immunomodulatory therapy has gained attention in recent years for treatment and management of immunocompromised individuals with chronic diseases.

**Fig. 1:**
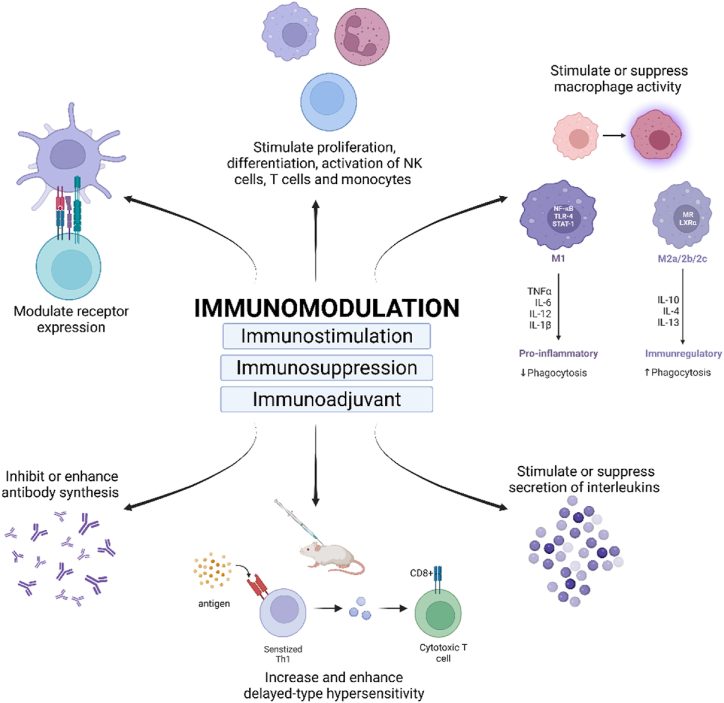
Immunomodulation mechanism of traditional plants. Immunomodulators either stimulate, suppress, or enhance various immune cells and signalling pathways to maintain immune homeostasis. Created with Biorender.com. Abbreviations – IL: interleukin, TNF-α: tumour necrosis factor-α, NK: natural killer cell, Th1: type 1 helper T cells.

The observation of specific weakened infectious agents can enhance the immune system to fight subsequent infections with the same or closely related infectious agents made by Edward Jenner through the discovery of the smallpox vaccine in 1796, led to the concept of “immunomodulation” [7]. The immune system of a healthy body maintains homeostasis inside the organism and protects it from invading pathogens such as bacterial, viruses, and allergens [7,8]. Moreover, endogenous or exogenous factors might influence the goal and efficiency of the immune system through immunosuppression or immunostimulation [8]. Substances that result in this activity are termed immunomodulators. They can be molecules of natural, artificial, or biological origins that could stimulate, suppress or modulate the innate and adaptive arms of the immune response [9]. Immunomodulators primarily target cellular responses (Fig. 1) such as protein synthesis, apoptosis, and antigen presentation, besides modulating transcription factors and mediators [8]. Based on clinical practice, immunomodulatory drugs or therapies are divided into immunosuppressors, immunostimulators, and immunoadjuvants [5]. Immunostimulators are genetically non-specific since it is envisioned as enhancing a body's resilience to infection either through innate or adaptive immune responses. Immunostimulators improve the resistance of the immune system to various chronic immunological disorder. They can also function preventative and booster agents, such as immunopotentiators, to boost the fundamentals levels of immune response while acting as therapeutic agent in individuals with impaired immunity [10]. Secondly, immunosuppressors play a viral ole in suppressing and restoring the immune system to its normal state when it fails to establish tolerance and becomes hyperactive [7]. They are commonly used to control the pathological immune response after organ transplantation by inhibiting immune system. Next, immunoadjuvants stimulate the immune system and elevate the response to vaccine without having any specific antigenic effect. A typical example of a commonly used immunoadjuvant is Freund's adjuvant used in the Bacillus Calmette-Guerin (BCG) vaccination [7].

Currently, chemically or biologically synthesized immunomodulatory drugs are widely used for disease treatment and prevention studies. Some examples include, corticosteroids, non-steroidal anti-inflammatory drugs, histamine antagonists, monoclonal antibodies, cellular signalling and cytokine inhibitors [11]. However, due to the adverse effects of these drugs posing significant health risks, immunomodulators isolated from traditional or medicinal plants with less severe side effects are now emerging as frontline treatment agents for cancer, infectious diseases and autoimmune disorders [12]. According to World Health Organization (WHO), a medicinal plant is defined as a plant, which, in one or more of its organs contains substances that can be used for therapeutic purposes, or which are precursors for chemo-pharmaceutical semi-synthesis [[13], [14], [15]]. The WHO has accounted about 60% of the world's population relying on traditional medicine and 80% of the population in the developing countries depend almost entirely on traditional medical practices, in particular, herbal remedies, for their primary healthcare. This rate can be said to be higher in developing countries [16]. The use of plants for therapeutic purposes is more preferable since plant materials are more affordable and accessible than synthetic drugs in these countries [16].

Herbal treatments have been utilized for millennia to cure a variety of ailments, however, there is still an ongoing debate on the origins of plant-based medications with pre-historical evidence documenting the use of plants for medicinal purposes as early as 60,000 years ago [16,17]. Moreover, writings in historical texts or holy books such as *Avesta*, *Bible*, *Talmud*, *Rig Veda*, *Atharva Veda*, *Pen T′ Sao*, *Canon of Medicine*, *Al*-*Hawi*, *Corpus of Simples*, *The Iliad* and *The Odyssey* indicate the use of plant-based medications in ancient Sumerian, Egyptian, Chinese, Indian, Jewish, Persian, Greek, and European civilizations [13,17,18]. At the moment, an estimated number of 35,000–70,000 plants species are used worldwide for medicinal purposes, with approximately 7000 and 6500 plants identified in South and Southeast Asian countries, respectively [19]. Current immunomodulatory therapy that focuses on the modulation of immune response has long been in practice of Ayurvedic medicine, under the concept of “Rasayana” [20]. Plants that have been grouped as Rasayanas typically have high nutritional content, antioxidant capacity and rejuvenating properties. In addition to their immunomodulatory effects, medicinal plants or herbs have anti-asthmatic, anti-arrhythmic, anti-inflammatory, hepatoprotective, hypercholesteraemic, antifungal, cardiotonic, diuretic and other medicinal activities [21]. They also have an antagonistic effect on oxidative stressors, decreasing the formation of various free radicals. Collectively, immunomodulators from traditional plants or herbs, boosts physical and chemical health, strengthens the body's defensive mechanism, hence, increases longevity [7,22].

Phytochemicals of medicinal plants, which were proven to regulate the cellular immune system, primarily mediate the immunomodulatory activity of medicinal plants. Phytochemicals are naturally found in fruits, vegetables, and medicinal plants. They are involved in a variety of reproductive and defensive mechanisms in plants such as controlling the odour and colour for protection and pollination, hormonal mechanisms for growth and developments, phytoalexins, and allelochemicals for pathogens and herbivorous defence [23]. A similar effect has been observed in humans, wherein immune response modulated by plant extract with either stimulatory or suppressive activities resulting in disease-free state individuals. Besides, various in vitro and in vivo models have reported the immunomodulatory properties of phytochemicals and plant derivatives such as polysaccharides, flavonoids, carotenoids, terpenoids, alkaloids, lipids, tannins, phenolic and aromatic compounds (Table 1) [3,22].

**Table 1:** Effects of plant-derived immunomodulators on the immune system.

Immunomodulatory action	Effect on the immune system	References
Immunostimulation	Increase lymphocyte and antibody production	[24]
	T cell proliferation and differentiation	
	The proliferation of NK cells	
	Monocyte proliferation, differentiation, and activation	
	Secretion of interleukin (IL)	
	Increase DTH response	
	Increase macrophage activity	
Immunosuppression	Decrease cytokine production	[24]
	Inhibit leukocyte	
	Inhibit antibody synthesis	
	Decrease activity of macrophages	
	Inhibit the production of iNOS	
Immunoadjuvant	Stimulate macrophage-monocyte system	[[25], [26], [27]]
	Enhance the role of helper-T cells	
	Promote proliferation of lymphocytes	
	Triggers activation of CD4^+^ and cytotoxic T cells	
	Increase antibody production	
	Induce DTH	
	Modulate receptor expression	

Abbreviations - NK cells: natural killer cells, IL: interleukin, iNOS: inducible nitric oxide synthase, DTH: delayed type hypersensitivity.

In this present review, authors provide a comprehensive overview of molecular targets and signalling pathways modulated by phytochemicals with known immunomodulating properties, specifically from *Curcuma longa* L. *Panax ginseng* C. A. Meyer, and *Moringa oleifera* Lam. Relevant articles related to immunomodulatory properties of these three plants were searched in ScienceDirect, Google Scholar, and Research gate databases with combination of keywords such as immunomodulators, plant immunomodulators, traditional or medicinal plants, synthetic or chemical synthesized immunomodulating drugs, *Moringa oleifera* Lam., *Curcuma longa* L, turmeric, curcumin, and *Panax ginseng* C. A. Meyer.

## 2 Traditional plants exhibiting immunomodulatory properties

**Fig. 2:**
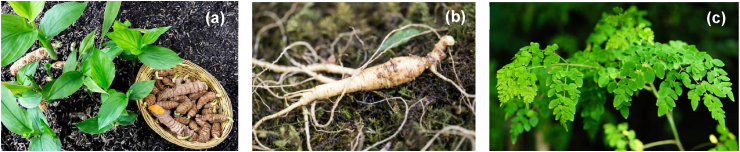
**Examples of traditional plants exhibiting immunomodulatory activities.** (a) *Curcuma longa.* L; (b) *Panax ginseng* C. A. Meyer. (c) *Moringa oleifera*. Lam (Picture courtesy of Google images) [[29], [30], [31]].

Numerous traditional plants with medicinal properties are known to have immunomodulatory properties alongside other pharmacological activities. Traditional plants have been demonstrated to maintain immune homeostasis by regulating multiple signalling pathways involved in inflammatory processes and oxidative stress responses that often lead to chronic inflammation, autoimmune diseases and cancer. The ability to communicate with a diverse range of immune mediators such as B and T lymphocytes, macrophages, natural killer (NK) cells, dendritic cells (DCs), cytokines, transcription factors and their downstream signalling pathways which are critical for immune response potentially contribute to immunomodulatory properties of traditional or medicinal plants [28]. In addition, various studies have demonstrated that immunomodulatory properties of phytochemicals from traditional plants correlate with their antioxidant, anti-inflammatory, anti-allergic, anti-proliferative, anti-tumour and anti-cancer properties. Despite extensive research, the precise immunomodulatory mechanism of most traditional plants have not yet been fully elucidated, justifying the need for further experimentation. Nevertheless, some traditional plants as shown in Fig. 2(a-c), specifically, *Curcuma longa* L, *Panax ginseng* C. A. Meyer, and *Moringa oleifera* Lam., have been appropriately studied for their role in regulating and modulating the immune system are described in the following sections.

### *2.1* *Curcuma longa.* L

#### 2.1.1 History of *Curcuma longa* L

*Curcuma longa* or commonly known as turmeric is a well-known traditional plant that belongs to the Zingiberaceae family. Turmeric is native to India and is widely cultivated in China, Sri Lanka, West and East Africa and other tropical countries. It is often referred to as the “Indian saffron or The Golden Spice of India” due to its use as a spice, food preservative and colouring source in Indian homes. Apart from being one of the oldest spices used in India, it also has a significant role in Siddha, Ayurveda, Chinese and Unani traditional medicinal systems for the treatment of various immunological disorders including cancer, psoriasis, inflammation, diabetes and ulcers. Evidence of the use of turmeric in Ayurveda were reported in Kusthagna (anti-dermatosis), Dashemani Lekhaniya (emaciating) and Visaghna (anti-poisonous) texts and also in Indian material medica (Dravyaguna Shastra). Meanwhile, in China, turmeric was being used to relieve urticaria, dermatitis, hepatitis infection, inflammatory joints, sore throat, and wounds [17,32].

#### 2.1.2 Phytochemical composition of *Curcuma longa* L

Generally, secondary metabolites such as alkaloids, tannins, flavonoids, glycosides, carbohydrates, terpenoids and phlobatannins are found in turmeric extracts. Nevertheless, composition of phytochemicals and percentage of yield may vary depending on polarity of solvent. Comparative studies between different solvents indicated that the ethanol extracts have a greater potential of extracting phytochemical that are biologically active. This was supported by study from Grover and colleagues where ethanol extracts recorded the highest percentage of yield and was found to be rich in phytochemical constituents such as alkaloids, carbohydrates, glycosides, saponins, steroids, proteins, terpenoids, flavonoids, anthraquinones, phlobatannins, and tannins as opposed chloroform extracts [33]. Additionally, in another study, coumarins, anthocyanins and reducing sugars were also detected in ethanol extracts of turmeric rhizomes [34]. Furthermore, ethanolic extracts have greater antioxidant properties due to its excellent radical scavenging activities which are associated with high levels of polyphenols and flavonoids [35]. Consequently, increasing concentration of ethanol, increased the content of flavonoids and polyphenols, however, reduced the percentage of yield in turmeric leaf extracts [36]. Saponins were abundantly found in petroleum ether extract of turmeric rhizome followed by phenols, alkaloids, flavonoids and tannins suggesting that the type of solvent used for extraction highly influence the phytochemical composition. Hence, the best extraction solvent can be varied depending on the extraction purpose and target parameter [35]. For example, ethanol extracts can be used for the purpose of extracting high polyphenols and flavonoids while water can be used to extract various bioactive compounds [36].

The use of turmeric in traditional medicine is supported by the presence of more than 300 biologically active components including polyphenols, sesquiterpenes, diterpenes, terpenoids, sterols, and alkaloids. However, the main special metabolites present in *Curcuma longa* L. belong to the sesquiterpene and diarylheptanoids classes. Major bioactive components isolated from turmeric are curcuminoids and volatile oils. Curcuminoids are phenolic compounds that are composed of curcumin (77%), bisdemethoxycurcumin (3%), demethoxycurcumin (17%) and cyclocurcumin [37]. Curcumin is chemically classified as a diarylheptanoid comprising of two aromatic rings, two hydroxyl and two methoxyl groups. Moreover, curcumin is a type of flavonoids that is hydrophobic and contributes to the yellow pigmentation observed in turmeric. Turmerone, curcuphenol, curlone are the main sesquiterpenes in turmeric with the concentrations of 30, 10.6 and 10% in the plant rhizomes respectively [37]. Other essential oils isolated from the turmeric rhizomes include, α-phellandrene, α-curcumene, β-bisabolene, β-sesquiphellandrene. Furthermore, α- and β-turmerones and aromatic turmerones contribute to the aroma of this spice [38]. Besides, turmerone also shows biological properties such as inhibition of platelet aggregation and antidiabetic.

#### 2.1.3 Regulatory mechanisms of *Curcuma longa* L

**Fig. 3:**
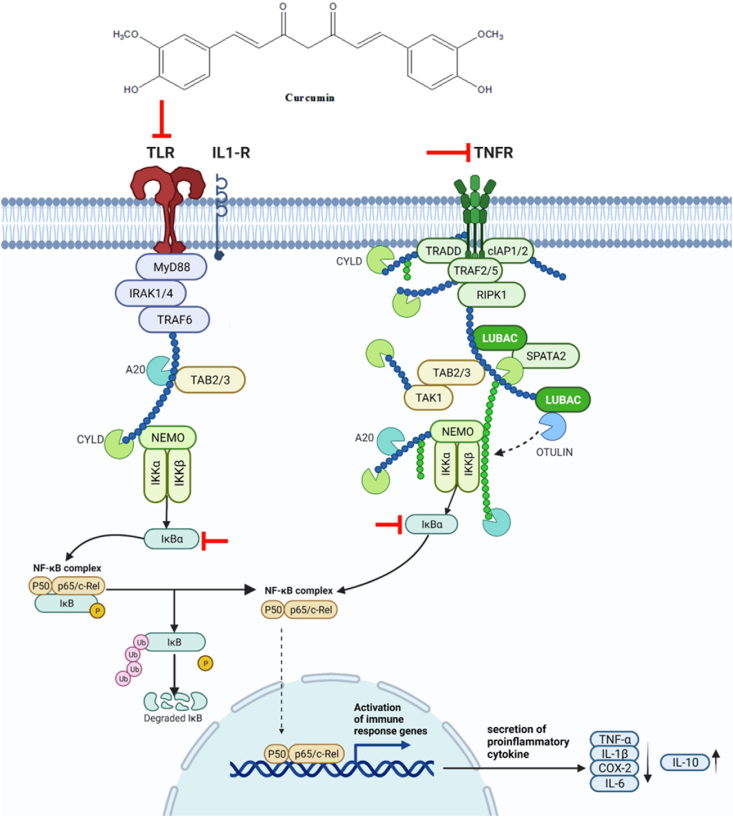
Immunomodulation mechanism of curcumin of *Curcuma longa*. L via NF-κB and TNF-α pathways. Created with Biorender.com Abbreviations – TLR: toll-like receptor, IL-1R: interleukin-1 receptor, TNFR: tumour necrosis factor receptor, MyD88: Myeloid differentiation primary response 88, IRAK: Interleukin-1 receptor-associated kinase, TRAF: tumour necrosis factor receptor–associated factor, SPATA: Spermatogenesis-associated protein 2, TRADD: Tumour necrosis factor receptor type 1-associated DEATH domain protein, RIPK1: Receptor interacting protein 1, TAB: TGF-beta activated kinase 1 (MAP3K7) binding protein 2, IκB: inhibitor of nuclear factor-κB, IKK: IκB kinase, NEMO: NF-kappa-B essential modulator, A20: A20 deubiquitinase, LUBAC: E3 ligase linear ubiquitin chain assembly complex, cIAP: Cellular inhibitor of apoptosis, TNF-α: tumour necrosis factor-α, COX-2: cyclooxygenase-2, IL: interleukin.

Due to its high medicinal value, *Curcuma longa* L. has become one of the most highly researched traditional plant from the *Curcuma* genus worldwide. Curcuminoids and its derivatives were reported to possess antioxidant, anti-inflammatory, anti-proliferative, anti-cancer, and immunomodulatory properties **(**Table 2**)**, thus making them suitable for the treatment of various autoimmune disorders including inflammatory bowel disease, allergy, diabetes, Alzheimer's disease, rheumatoid arthritis (RA), and multiple sclerosis [6,39,40]. Curcumin remarkably regulates the production of reactive oxygen species (ROS) and nitric oxide (NO) and secretion of pro-inflammatory mediators such as interleukin-(IL), IL-1β, IL-2, IL-6 and IL-10 that mediate intracellular signalling pathways such as nuclear factor kappa B (NF-κB), mitogen-activated protein kinases (MAPKs), Activation protein (AP-1), and the Notch-1 pathway involved in inflammation (Fig. 3) [[41], [42], [43]].

**Table 2:** Immunomodulatory action of *Curcuma longa* Lam.

Plant part/Solvent	Isolation of compounds/Major compound	Study Design	Immunomodulatory mechanism	Biological activities	Ref
**Ethanolic extract of turmeric rhizome**	apigenin 7-O-α-l-rhamnoside-4′-O-β-D-glucoside, 7-methoxyapigenin-6-C-β-d-glucopyranoside, N-(3-methoxyphenyl) acetamide	Streptozotocin induced Sprague-Dawley white albino mice	↓ IL-6, IL-1β, and TNF-α ↓ total IgE counts ↓ total leukocyte count	Anti-inflammatory Antioxidant	[44]
**Curcumin extract**	Curcumin	Caecal ligation and puncture-induced C57BL/6 model	↓ TNF-α, IL-1β, IL-6, and IL-17-A ↓ mRNA and nuclear translocation of p65 ↑ IL-2 and IL-10 ↑ macrophage polarization from M1 to M2 ↑ rate of regulatory T lymphocytes	Immunomodulation	[45]
**Curcumin extract**	Curcumin	DSS-induced colitis in BALB/c mice	↑ Regulatory T lymphocytes ↓ IL-1β, IL-2, IL-6, IL-9, and IL-17A ↓ PI3K, p-PI3K, Akt, pAkt, c-Myc ↓ polarization of inflammatory DCs ↓ Th17 cells	Immunostimulation	[40]
**Curcumin extract**	Curcumin	carbon tetrachloride-induced hepatic cirrhosis in *Rattus rattus* L. rats	↓ TNF-α, and TGF-1β ↑ IL-10	Anti-inflammatory	[46]
**Curcumin extract**	Curcumin, bis-demothxy-curcumin, demothxy-curcumin	Ovalbumin-sensitized Wistar rats	↓ total WBC, eosinophil, neutrophil, and monocyte ↓ Total IgE ↓ PLA2, IL-4, NO_2_, NO_3_ ↑ balance of Th1/Th2 cells ↑ IFN-γ and IFN-γ/IL-4 ratio	Anti-inflammatory Antioxidant Immunomodulation	[47] [48]
**Ethanolic extract of turmeric rhizome**	Curcumin	LPS-stimulated RAW 264.7 macrophage cells	↓ NO production ↓ ERK1/2, pERK1/2, p38	Anti-inflammatory	[48]
**Ethanolic extract of turmeric rhizome**	Curcumin	cyclophosphamide-induced immunosuppression C57BL/6 mouse models	↑ NK cell activity	Immunostimulation	[48]
**Aqueous extract of turmeric rhizome**	Curcumin	C57/B1 male mice	↑ T lymphocyte proliferation	Immunostimulation	[49]
**Ethanolic extract of turmeric rhizome**	Curcumin	Ovalbumin-sensitized Balb/c mice	↓ Total IgE count ↓ IgG1 levels ↑ IgG2a levels ↑ IFN-γ, TGF-β1 ↓ IL-4, IL-5, and IL-13 ↓ Th1- and Th2-related cytokines, IL-17 production, and Th17-related cytokine	Immunomodulation	[50]
**Ethanolic extract of turmeric rhizome**	Curcumin	Ovalbumin-sensitized asthmatic rats	↓ total WBC count, neutrophil, eosinophil, ↓ PLA2, IgE, NO_2_, and NO_3_ ↑ lymphocyte count, IFN-γ/IL-4 ratio, and IFN-γ,	Immunomodulation	[51]
**Essential oils from turmeric rhizome**	NBFR-03, turmerone, aromatic turmerone	Nude mouse human cervical cancer xenograft model	↓ tumour volume	Anti-proliferative	[52]
**Aqueous extract of turmeric rhizome**	β-turmerone, α-turmerone, Ar-turmerone, 16-kauren-19-yl acetate, α-atlantone, β-sesquiphellandrene, benzene	LPS-stimulated RAW 264.7 macrophage cells	↑ IL-10 ↓ TNF-α, IL-4 and IL-6 ↓ NO production	Anti-inflammatory	[53]
**Butanol extract of aerial parts of turmeric roots**	Quercetin 3-(2Gal-rhamnosyl-robinobioside), Quercetin 3-rutinoside, Quercetin 3-rhamnosyl-(1 → 2)-rhamnoside, Quercetin-3-O-rhamnoside	LPS-stimulated RAW 264.7 macrophage cells	↓ NO, iNOS and COX-2 production ↓ TNF-α and IL-6 ↓ IκBα, p65, p50	Anti-inflammatory	[54]
**Ethanolic extract of turmeric root**	modified pectic polysaccharide, MTrPP	*H. pylori* induced inflammatory stimulus for gastric ulceration In Wistar rats	↓ NO production, COX-2, p65, TNF–α, IL–8, *p*-ERK1/2, galectin-3, MMP-9 ↑ IL-10, COX-1, PGE_2_, p-p38	Anti-inflammatory	[55]
	Calebin A	Jurkat, U937, A293, HCT116, H1299, MCF-7, SCC-4, RPMI 8226, MM.1S, U266 cell lines	↓ TNF–α, p65, COX-2, Bcl-2, c-IAP-1, cFLIP, XIAP, cyclin D1, c-Myc, ICAM-1, VEGF ↑ cleavage of caspase-3, -8, and -9	Anti-inflammatory	[56]
	Curcumin	DSS-induced colitis in C57BL/6 mice	↓ CCL17, CXCL5 ↑ IL-10, IL-11, FoxP3, regulatory T lymphocytes	Anti-inflammatory	[57]
	Curcumin, bis-demothxy-curcumin, demothxy-curcumin, reduced curcumin	LPS-stimulated RAW264.7 macrophage cells HeLa cells	↓ pIKKβ, p-p65, degradation of IκBα	Anti-inflammatory	[58]
**Ethanolic extract of turmeric rhizome**	curcumin, α-turmerone, β-turmerone, aromatic turmerone	HT29 and HCT116 cells	↓ cell proliferation by α/β-turmerone and curcumin but not aromatic turmerone	Anti-proliferative	[59]
**Hot water extract of turmeric rhizome**	tumeronol A and turmeronol B	HUVEC cell	↓ PGE_2_, NO, COX-2 mRNA, iNOS mRNA, IL-6, IL-1B, TNF-a, nuclear translocation of p65	Anti-inflammatory	[60]
**Ethanolic extract of turmeric rhizome**	curcumin, α-turmerone, β-turmerone, aromatic turmerone	HT29 xenograft model	↓ tumour growth by curcumin (26.65%) and turmeric extract (38.9%) ↓ CD31 ad VEGF-R2 endothelial cell markers	Anti-angiogenic	[59]

↓: decrease/reduced, ↑: increased/elevated.

Abbreviations – IL: interleukin, TNF-α: tumour necrosis factor-α, IgE: immunoglobulin E, IgG: immunoglobulin G, mRNA: messenger ribonucleotide, PI3K: phosphoinositide 3-kinase, pPI3K: phosphorylated PI3K, Akt: Protein kinase B, pAkt: phosphorylated Akt, c-Myc: cellular myelocytomatosis oncogene, DC: dendritic cells, Th1: type 1 helper T cells, Th2: type 2 helper T cells, Th17: type 17 helper T cells, WBC: white blood count, PLA2: phospholipase 2, NO_2_: nitrites, NO_3_: nitrate, NO: nitric oxide, ERK1/2: extracellular signal-regulated kinase, pERK1/2: phosphorylated extracellular signal-regulated kinase, NK: natural killer cell, IFN: interferon, TGF-β: transforming growth factor-β, iNOS: inducible nitric oxide synthase, COX: cyclooxygenase, COX-2: cyclooxygenase-2, IκBα: inhibitor of nuclear factor-κB-α, MMP-9: Matrix metalloproteinase-9, PGE: prostaglandin, Bcl-2: B-cell lymphoma 2, cIAP: Cellular inhibitor of apoptosis, ICAM: intercellular adhesion molecule, cFLIP: Cellular FADD-like IL-1β-converting enzyme, XIAP: X-linked inhibitor of apoptosis protein, CXCL-5: chemokine (C-X-C motif) ligand-1, CCL-17: CC chemokine ligand 17, VEGF: Vascular endothelial growth factor, DSS: dextran sulphate sodium.

Growing evidence indicate that modulation of NF-κB pathway is one the critical targets of curcumin. Curcumin modulates the NF-κB pathway by inhibiting the phosphorylation of I kappa B kinase -α (IKKα) and preventing the nuclear translocation of NF-κB p65 subunit suggesting that direct modulation of curcumin of NF-κB network and its activation process. In a study conducted by Tyagi et al. (2017), Calebin A, a polyphenolic compound isolated from curcuminoids significantly inhibited TNF-α induced NF-κB activation in various cancer, lymphoma and leukaemia cell lines thus contributing to its anti-inflammatory and anti-cancer properties. Calebin A primarily suppresses the activation of NF-κB by blocking the binding of p65 to DNA. Interestingly, Cys38 mutation in p65 abrogated suppressive effect of Calebin A although no effect of was observed in the binding of mutated p65 to DNA indicating that Calebin A targets Cys38 in NF-κB p65 subunit. Furthermore, partial suppression of inhibitor of NF-κB alpha (IκBα) degradation by Calebin A indicates the potential involvement of other signalling pathways in inhibition of NF-κB activation. Similarly, TNF-α stimulated HeLa cells when treated with curcumin and commercial turmeric extract (curcumin, bisdemethoxycurcumin, and demethoxycurcumin) inhibited NF-κB activity by suppressing IKKβ and NF-κB p65 phosphorylation and IκBα degradation [58]. Likewise, turmeronol A and B also prevented NF-κB activation by inhibiting nuclear translocation of NF-κB p65 in LPS-stimulated RAW264.7 murine macrophage cells [60]. Besides, butanol fraction of aerial parts of Curcuma longa consisting of flavonoids also exert anti-inflammatory effects by inhibiting the phosphorylation and degradation of IκB, nuclear translocation of NF-κB p65 and p50, thus, preventing activation of NF-κB [54].

Several NF-κB associated pro-inflammatory cytokines expressions, such as IL-1, IL-2, IL-6, IL-8, IL-12 and TNF-α, are shown to be downregulated following treatment with curcumin. By contrast, curcumin or turmeric extract or both, stimulates IL-10, an anti-inflammatory cytokine, to counteract inflammatory responses and production of pro-inflammatory cytokines such as IL-1, interferon gamma (IFN-γ) and IL-2 from T lymphocytes contributing to its as anti-fibrotic, anti-inflammatory and immunomodulatory properties [46,61]. Increased IL-10 expression in upregulation of IL-10 and IL-11 anti-inflammatory cytokines through upregulation of regulatory T cells were observed in dextran sodium-sulphate -induced ulcerative colitis mice models upon combined treatment of turmeric essential oils [57]. Similarly, curcumin treatment protects Caecal ligation and puncture-induced acute lung injury mice model by regulating IL-10 production via regulatory T cells indicating that regulatory T cells are important molecular target of curcumin so that it can exert modulatory immune responses via IL-10 [45].

Oxidative stresses caused by increase in ROS production and NO secretion leads to activation of NF-κB and tumour necrosis factor (TNF)-α pathways which are crucial in mounting inflammation [62,63]. Antioxidant and anti-inflammatory properties of curcumin were reported due to its ability to inhibit ROS generating enzymes such as lipoxygenase (LOX), cyclooxygenase (COX), and inducible nitric oxide synthase (iNOS). Evidently, components of arachidonic acid metabolic pathway which primarily involve phospholipase enzyme A2 (PLA2), LOX, COX-1, COX-2 and pro-inflammatory prostaglandins, specifically prostaglandins E2 (PGE_2_) are targeted by curcumin for immunosuppression. Curcumin inhibits the generation of arachidonic acid by preventing the phosphorylation of PLA2 enzyme and enzymatic reactions of COX [41]. In the arachidonic acid pathway, the PLA2 enzyme is responsible for the hydrolysis of membrane phospholipids and release of arachidonic acids while COX enzymes are responsible for the conversion of arachidonic acid into pro-inflammatory PGE_2_ [41]. To support this statement, reduction in PLA2 levels followed by suppression of lung inflammation were observed in ovalbumin sensitized mice upon treatment with curcumin indicating the potential role of curcumin as a therapeutic agent in allergic asthma [[64], [65]]. Various pathological conditions, including RA and cancer, result from the incomplete metabolism of AA or accumulation of PGE_2_. Curcumin suppressed the PGE_2_ production in synovial fibroblasts of RA patients by inducing apoptosis [66].

Immunomodulatory properties of curcumin or turmeric extract have been observed in several in vitro and in vivo animal models. Curcumin or turmeric extract modulate immune responses related to helper T (Th)- 1, Th2 and Th17 related cytokines, in addition to modulating the proliferation of different subpopulation of T lymphocytes [[44], [49], [50]]. Curcumin was reported to attenuate allergic airway inflammation by inhibiting NF-κB and its downstream transcription factor, goblin transcription factor 3 (GATA3) [62,67]. Balb/c mice models investigating the anti-inflammatory properties of curcumin reported that curcumin improved airway inflammatory cell infiltration and downregulated the Notch1/2 receptors and GATA3 signalling pathways, preventing the development of allergic airway inflammation [62,63,67]. Moreover, curcumin demonstrated an immunosuppressive and immunoregulatory effect by modulating Th1/Th2 balance [68]. Allergic responses are also controlled by curcumin by attenuating Th2 inflammatory reactions [68]. Studies reported that NF-κB was required for GATA3 expression and Th2 differentiation. Moreover, studies have identified that the GATA3 promoter directly targets the Notch signalling, confirming that GATA3 and NF-κB are required in Notch-induced Th2 differentiation [62,63,67]. Furthermore, Th1/Th2 balance was also modulated when ovalbumin-sensitized mice exhibiting food allergies showed reduced expression in Th2 related cytokines such as IL-4, IL-5 and IL-13 while increased expression of Th1 related cytokines such as IFN-γ upon curcumin treatment [50]. Similar results were also obtained in another study of allergic asthma indicating that curcumin modulates immune responses from Th2 to Th1 dominant response by stimulating Th1 and suppressing Th2 cells [50,47,51]. Reduction of IL-17 expression and Th17 cells, elevation in regulatory and follicular T cells were observed in various in vivo mice models that representing pathophysiology allergic and inflammatory diseases such asthma, gastric inflammation and food allergy [50,47,51].

### *2.2* *Panax ginseng* C.A. Meyer

#### 2.2.1 History of *Panax ginseng* C. A. Meyer

*Panax ginseng* is another type of traditional plant known to exhibit immunomodulatory properties. *Panax ginseng* or commonly known as the Asian ginseng, Chinese ginseng, or Korean ginseng belongs to the Araliaceae family that includes other common *Panax* species such as *Panax quinquefolius* L. (American ginseng), *Panax japonicus* C. A. Mey (Japanese ginseng), and *Panax notoginseng* (Burk.) f. H. Chen, (notoginseng). Despite its common name, each species of Panax is distinct and vary in phytochemical composition and functions. Among these commonly known *Panax* species, *Panax ginseng* C. A. Meyer is the most commonly used herbal medicine in China and other Asian countries for the treatment and management of various ailments and diseases [69].

#### 2.2.2 Phytochemical composition of *Panax ginseng* C. A. Meyer

Generally, *Panax ginseng* possesses various chemical components such as ginsenosides, polysaccharides, polyacetylenes, proteins, lipids, peptides, amino acids, organic acids, vitamins, and fat-soluble molecules [70]. In this review, much focus has been given to the polysaccharides and saponin of ginseng as they are the main bioactive compounds isolated from ginseng. Saponins of ginseng are called ginsenosides. They are dammarane triterpenoids saponins that can be divided into two species according to protopanaxadiol and panaxatriol aglycone groups. Protopanaxadiol contains ginsenosides Ra1, Ra2, Ra3, Rb1, Rb2, Rb3, notoginsenosides R4, Rs1, Rs2, Rs3, Rs4, and malonyl ginsenosides Rb1, Rb2, Rc and Rd and compound K ginsenosides, while panaxatriol groups contain Re, rf, Rg1 and notoginsenosides R1 [71,72]. The main components of ginseng polysaccharides (GPs) are arabinogalactan, pectin and acidic polysaccharides mainly consisting of (1 → 6)-glycosidic linkages and (1 → 3)-glycosidic linkages. Meanwhile, the monosaccharides of ginseng are mainly composed of l-arabinose, d-galactose, l-rhamnose, d-galacturonic acid and d-glucuronic acid [73].

#### 2.2.3 Regulatory mechanism of *Panax ginseng* C. A. Meyer

**Fig. 4:**
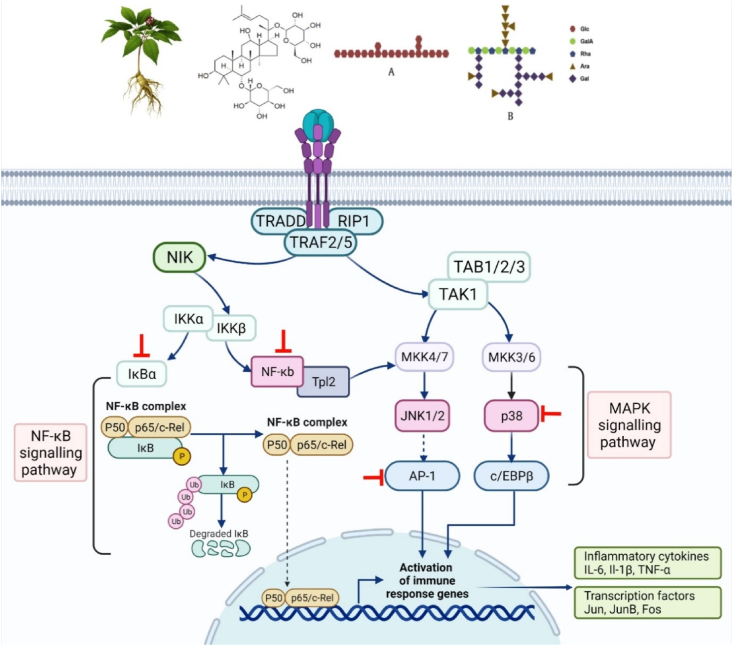
Immunomodulatory mechanism of action of *Panax ginseng* C. A. Meyer. The mechanism of action involves NF-κB, MAPK, and JNK pathways. Created with Biorender.com. Abbreviations –TNFR: tumour necrosis factor receptor, TRAF: tumour necrosis factor receptor–associated factor, TRADD: Tumour necrosis factor receptor type 1-associated DEATH domain protein, RIP1: Receptor interacting protein 1 TAK: TGF-β-activated kinase 1, MAPK: Mitogen-activated protein kinases, JNK: c-Jun N-terminal kinase, MKK: mitogen-activated protein kinase (MAPK) kinase 4, AP-1: activator protein-1: Fos, AP-1 Transcription Factor Subunit, Tpl2: Tumour progression locus 2, IκB: inhibitor of nuclear factor-κB, IKK: IκB kinase, NEMO: NF-kappa-B essential modulator, TNF-α: tumour necrosis factor-α, COX-2: cyclooxygenase-2, IL: interleukin.

Ginsenosides or ginseng polysaccharides (GP) exert immunostimulatory and immunosuppressive activities on the immune system and can be used as an immunoadjuvant in vaccines. Ginsenosides or GP regulate many different pathways, including MAPKs (Fig. 4). They are known to have promising pharmacological activities such as antitumour, anti-inflammatory and immunomodulatory actions against various immunological disorders. Several reports suggests that ginsenosides or GP regulate many different pathways including MAPK and NF-κB mediated signalling **(**Table 3**)**. MAPKs are intracellular enzyme that are important in the stimulation of various immune responses, including stimulation of inflammatory cytokines, which will trigger GTPase stimulation of upstream kinases called mitogen-activated protein kinase kinases [74]. It involves a stepwise activation path via signalling enzymes comprising of p38, extracellular signal-regulated kinase (ERK), and Jun N-terminal kinase (JNK) which regulate diverse range of cellular functions such as proliferation, activation and degranulation. Phosphorylation of serine/threonine kinases followed by activation of MAPK, results in the phosphorylation of p38 MAPK. p38 MAPK is involved in transcription-dependent and post-transcriptional control mechanisms that produce inflammatory mediators. MAPKs activate NF-κB pathway, whose translocation into the nucleus initiates the transcription of inflammatory and allergy-related mediators [74].

**Table 3:** Immunomodulatory action of *Panax ginseng* C. A. Meyer.

Plant extract	Isolation of compounds/Major compound	Study Design	Immunomodulatory mechanism	Properties	Ref
**Methanol extract of roots (APSF)**	G-Rg1, G-Re, G-Rb1, G-Rc, G-Rb2, and G-Rd	LPS-stimulated RAW264.7 murine macrophages	↓ NO production, mRNA iNOS, COX-2, TNF-α, c-Jun, p-p38, MKK3/6, TAK1,	Anti-inflammatory	[75]
**Ethanol extracts of roots (BIOGF1K)**	a compound K-rich fraction	LPS-stimulated RAW264.7 murine macrophages, HEK293 cells	↓ NO production, mRNA of iNOS and IFN-β, MyD88, TRIF, *p*-IκBα, *p*-IKKα/β, pAkt, pIRF3, *p*-TBK1	Anti-inflammatory	[76]
**Methanol extract of root (TSG)**	NA	LPS-stimulated RAW264.7 murine macrophages	↓ NO production, iNOS, TNF-α, IL-1β, NF-κB p65, degradation of IκBα, p-p38 MAPK, *p*-ERK, *p*-JNK	Anti-inflammatory	[77]
**Root extract**	ginsenoside Rb1	LPS-stimulated RAW264.7 murine macrophages, BMDMs, THP-1	↓ NO, nitrite, iNOS, COX-2, ROS, Ca2+, TNF-α, IL-6, IL-1β, NF-κB, *p*-IKK-α/β, nuclear translocation of p65, pJNK1/2, p-p38 MAPK, MyD88, TAK-1, *p*-TAK1, and LPS-induced TLR4 dimerization,	Anti-inflammatory	[78]
**Root extract**	ginsenoside Rf	TNF-α or LPS-stimulated HT-29 cells or RAW264.7 cells	↓ NO, ROS, TNF-α production ↓ IL-1β, IL-6, and iNOS genes ↓ NF-κB, IKK, p65 nuclear translocation, p-38 phosphorylation, ERK, and JNK	Anti-inflammatory	[79]
**Root extract**	ginsenoside Ro	LPS-stimulated RAW264.7 cells	↓ TLR4, pIκBα, p65 cytosol, p65 nuclear translocation, ↓ pJNK, p-p38, *p*-ERK, p-*c*-Jun, p-*c*-Fos, *p*-ATF2	Anti-inflammatory	[80]
**Root extract**	ginsenoside Rc, Rb1, Rb2	LPS-stimulated RAW264.7 cells, TNF-α/IFN-γ-treated synovial cells, HEK293 cells	↓ TNF-α, nuclear translocation of *p*-ATF-2 and *p*-FRA-1, AP-1, *p*-IRF3, pTBK1, pIKKε, AKT	Anti-inflammatory	[81]
**RGSF of root extracts**	ginsenoside Rg1, Re, Rf, Rb1, Rc, Rb2, Rb3, Rd, F2, and Rg3	LPS-stimulated RAW264.7 cells HEK293 cells	↓ NO and PGE_2_ production ↓ iNOS, COX-2, TNF-α and IL-1β ↓ *p*-ERK, p-p38, *p*-MMK3/6, *p*-MEK1/2, AP-1 ↓ IκBα, *p*-IKKα/β, NF-κB	Anti-inflammatory	[82]
**Root extract**	ginsenoside Rb3, Rg3	RAW264.7 murine macrophage cells and THP-1 monocyte cells	↓ NO, PGE_2_, iNOS, COX-2, IL-1β, IL-6, and TNF-α ↓ p65 and IκBα ↓ *p*-JNK, p38 and *p*-ERK	Anti-inflammatory	[83]
**Ethanolic extract of root**	Neutral polysaccharides RGP1 - arabinose, glucose, and galactose (0.02:0.88:0.10) RGP2-1 - rhamnose, arabinose, glucose, and galactose (0.02:0.10:0.77:0.11)	LPS-stimulated RAW264.7 cells	↓ NO production, ↓ phagocytic activity ↑ TNF-α	Immunomodulation	[84]
**Root extract**	ginsenoside Rb2	cyclophosphamide-induced immunosuppression in Balb/c mice	↑ body weight, splenic weight, splenic B and T lymphocytes, NK activity ↑ IFN-g, IL-2, TNF-α, IgG ↑ IL-4, TNF-α, IL-2, SYK, and IL-6	Immunomodulation	[85]
**FGEP of root extract**	Pectin polysaccharide - rhamnose, arabinose, galactose, glucose, and galacturonic acid (FGEP-C - 2.1:10.5:10.4:62.2:14.3), (FGEP-A - 1.8:10.9:12.6:50.9:23.1), and (FGEP-CA - 3.2:11.4:16.5:45.8:22.3)	RAW264.7 murine macrophage cells	↑ IL-6, IL-12, and TNF-α	Immunomodulation	[86]
**FGWP from roots**	rhamnose, arabinose, galactose, glucose, and galacturonic acid (1.8:10.1:9.2:60.6:17.8)	Cy-induced immunosuppression in Balb/c mice	↑ spleen and thymus indices, splenic T and B lymphocytes, WBC count, and NK cell activity ↑ IL-2, IL-6, and IFN-γ	Immunomodulation	[86]

↓: decrease/reduced, ↑: increased/elevated.

Abbreviation – IL: interleukin, NK: natural killer, WBC: white blood cell, IFN: interferon, TNF-α: tumour necrosis factor-α, Ig: immunoglobulin, SYK: Spleen tyrosine kinase, NO: nitric oxide, LPS: lipopolysaccharide, PGE_2_: prostaglandin 2, COX: cyclooxygenase, IκBα: inhibitor of nuclear factor kappa B, JNK: Jun N-terminal kinase, *p*-JNK: phosphorylated JNK, ERK: extracellular signal-regulated kinase, *p*-ERK: phosphorylated ERK, NF-κB: nuclear factor kappa B, MKK: mitogen-activated protein kinase (MAPK) kinase, MEK: Mitogen-activated protein kinase kinase, ATF-2: activating transcription factor 2, MyD88: Myeloid differentiation primary response 88, TRIF: Toll/interleukin-1 receptor, ROS: reactive oxygen species, TAK: TGF-β-activated kinase 1, AP-SF: ginsenoside-enriched fraction, TSG: total saponin from ginseng, FGWP: hot water-extracted polysaccharide, FGEP: enzyme-assisted extracted polysaccharide, KRG: Korean red ginseng, RGSF: KRG saponin fraction.

The first line of defence in the immune system is provided by innate immune cells such as macrophages and neutrophils. Although there are two subpopulations of macrophages, namely, M1 and M2 macrophages, M1 macrophages play an important role in the inflammatory processes as these macrophages are stimulated by invading microorganisms such as lipopolysaccharide (LPS) or Th1 cytokines such as TNF-α and IFNγ [60]. Furthermore, they are characterised by their ability to upregulate pro-inflammatory genes such as IL-1β, IL-6, TNF-α, iNOS and COX-2 which often function in the onset inflammatory disease progression [60]. Methanol extract of ginsenoside-enriched fraction, AP-SF, BIOGF1K, a compound K rich fraction, total saponin ginseng (TSG), Rb1, Rf, Korean Red Ginseng Fraction (RGSF), and GRb3 inhibited LPS-stimulated RAW264.7 cells via suppressing pro-inflammatory genes IL-1β, IL-6, TNF-α, iNOS and COX-2 [[75], [76], [77], [78], [79], [82], [83]]. Upregulation of these genes are often associated with activated M1 macrophages. Therefore, targeting these molecules may potentially inhibit inflammation and inflammatory related diseases.

Numerous studies have reported that ginsenosides and GPs exert anti-inflammatory effects by modulating the MAPK and NFKB pathway. The translocation of NFKB dimers into the nucleus sets motion to initiate the transcription of inflammatory and allergy-related mediators. Specifically, components such as p38, ERK1/2, Activating transcription factor 2 (ATF-2), p65/p50, IKKα/β, and IκBα from MAPK and NF-κB pathways are targeted by GPs and ginsenosides. In normal conditions, the NF-κB exists as an inactive heterodimer consisting of three subunits namely, p50, p65 and IκB in the cytosol. However, LPS stimulation of RAW264.7 murine macrophages reported increased degradation of IκB kinases followed by translocation of NF-κB p65 subunit to the nucleus [77]. Nevertheless, when cells were treated with TSG extract, ginsenosides Rb1 and Ro, LPS-stimulated NF-κB activation were suppressed by inhibiting the degradation of IκBα, nuclear translocation of NF-κB p65 subunit from cytosol and expression of pro-inflammatory cytokines [77,78,80]. Binding of LPS to toll-like receptor-4 (TLR4) activates the NF-κB pathway and its downstream effector molecules. This ligand-receptor interaction is targeted by ginsenoside Ro and GRb3 which demonstrates dose-dependent suppression of TLR4 expression and binding of cell membrane to LPS indicating that ginsenosides directly target TLR4. Molecular docking studies have revealed that several ginsenosides including Ro, GRb3, competitively bind to the LPS/MD2 complex to inhibit TLR activation, which subsequently regulating the TLR4/NF-κB/MAPK inflammatory signalling [83,80]. In a study conducted by Mitra et al. (2022), it was reported that Korean red ginseng (KRG) water extracted prevented the phosphorylation of p38, ERK, and JNK through MAPK and NF-κB pathways in HaCaT and HMC-1 cell types, subsequently improving atopic-dermatitis like skin lesion [87]. Furthermore, TNF-α/IFN-γ induced inflammation in HaCaT cells when treated with Rg5:Rk1 suppressing NF-κB, p38 MAPK phosphorylation and NF-κB translocation levels. Similarly, AP-SF also increased the suppression phosphorylated p38, an upstream signalling enzyme that activates c-Jun [75,88]. Similarly, ginsenoside Ro has been shown to inhibit TLR4-induced activation of NF–B and MAPK signalling pathways in vivo and in vitro by inhibiting nuclear translocation of the p65 subunit and suppressing JNK, p38 and ERK phosphorylation levels in a dose-dependent manner [80]. Interestingly, ginsenoside Rc did not prevent MAPK phosphorylation, but p38 was reported to be a target of ginsenoside as it suppressed the phosphorylation of ATF2 [81]. Apart from ginsenosides, several reports indicated that ginsan, a polysaccharide extracted from Panax ginseng, exhibited immunomodulatory properties by inhibiting the NF-κB pathway and p38 MAP kinase [89].

Both GP and ginsenosides are involved in macrophages and enhancing their phagocytic activity. In a study conducted by Lee et al. (2022), it was reported that KRG saponin fraction suppressed NF-κB and Activator protein 1 (AP-1) transcription factors such as c-Jun, c-Fos and phosphorylated ATF2 during macrophage-mediated inflammatory responses in RAW264.7 cells. The suppression of the transcription factors indicates that KRG saponin exerted anti-inflammatory activities through immunosuppression. Moreover, TNF-α produced by macrophages in the synovial cells of rheumatoid arthritis patients was suppressed by ginsenoside Rc by suppressing the AP-1 activation [81]. Panax polysaccharides such as WGFPM, RGP1/2, WGPA-2-RG, GMP, WPS-1/2, and SPS-1/2/3 enhance phagocytic activity by activating macrophages [70]. Ginseng polysaccharides boost immunity by enabling stimulation of DC, which in turn promotes CD4^+^ T lymphocyte proliferation and CD86 expression [90]. The acidic portion of polysaccharides containing arabinogalactan (AG), type-1 rhamnogalacturonan (RG-I), and homogalacturonan (HG)-rich pectins can down-regulate immunostimulation by increasing the number of CD25^+^ immunoregulatory T cells, indicating a therapeutic effect on autoimmune diseases [91].

### *2.3* *Moringa oleifera* Lam

#### 2.3.1 Description of *Moringa oleifera* Lam

*Moringa oleifera* Lam. is another traditional plant with high immunomodulatory activity used in the Ayurvedic and Unani systems of medicine. According to taxonomical distribution, *Moringa oleifera* Lam. is one of the 13 species belonging to the family Moringaceae and its sole genus *Moringa*. Traditional names of *Moringa oleifera* Lam. vary according to geographical location. For instance, the plant is referred to as Shigru (Sanskrit), Drumstick or Horseradish Tree (English) or Kelor tree (Malay). The plant is native to India but is now widely cultivated in different regions including tropical and subtropical regions of the world as it holds remarkable health and environmental benefits. The plant is also referred to as the Miracle Tree due to its ability to survive in harsh conditions, high medicinal and nutritional properties and its industrial applications [92].

#### 2.3.2 Phytochemical composition of different parts of *Moringa oleifera* Lam. Tree

Almost all parts of the tree including leaves, flowers, seeds, and fruits are edible sources of food with high nutritional and medicinal values. Typical secondary metabolites isolated from this traditional plant include a diverse range of alkaloids, flavonoids, saponins, tannins, vitamins, polyphenols, polysaccharides, isothiocyanates, glycosides, fatty acids and essential amino acids. Despite the tremendous nutritional and medicinal properties, only leaves, seeds and roots are given major attention in research due to high phytochemical composition.

Phytochemical analyses comparing different parts of plants have demonstrated that leaves contain the highest quantity of phytochemical composition. Phytochemical analysis of different leave extracts such as methanol, n-hexane, aqueous, ethyl acetate and butane revealed the presence of alkaloids, tannins, flavonoids, phenolics and saponins in all extracts. Phytochemical constituents that are majorly isolated from leaves are glycosylated flavonoids quercetin-3-O-glucoside (isoquercitrin), kaempferol-3-O-glucoside (astragalin), quercetin-3-O-rutinoside (rutin), flavonoids (epicatechin, myricetin, rutin), phenolic acids (caffeic acid, syringic acid, gallic acid, sinapic acid), alkaloids (N,α-l-rhamnopyranosyl vincosamide, marumoside A, marumoside B), nitril glycoside (niazirin). Moreover, seed extracts of MO have the second highest phytochemical composition following leaves. Bioactive compounds such as fatty acids (oleic acid, palmitic acid, behenic acid, stearic acid), glucosinolates (glucomoringin, 3-Hydroxy-4-(α-*l*-rhamnopyranosyloxy) benzyl glucosinolate), saccharides (Cellotetraose, sucrose, cellotriose) were isolated from seed extracts of MO. Glucosinolates, is another type of secondary metabolite that can be isolated different parts of MO tree. The major compound of glucosinolates isolated in MO is glucomoringin (4-(α-L-rhamnopyranosiloxy) benzyl glucosinolates) can be isolated from stems, pods, leaves and seeds. Additionally, Glucomoringin can be enzymatically converted into an isothiocyanate called moringin, with the aid of an enzyme called, myrosinase [93]. Meanwhile, glucotropaeolin, a type of benzyl glucosinolates is majorly isolated from the roots of MO tree [[94], [95], [96], [97]].

Variations in the presence or absence of phytochemicals in several studies could be attributed difference in polarity, type of solvents used [95], method of extraction, period and geographical location of sample collection [98,99]. For instance, cell-based assay, namely, Folin-Ciocalteu method, used for quantification of total phenolic and flavonoid contents showed significantly higher total phenolic content and total flavonoid content in the aqueous extracts of MO leaves as opposed stems, twigs, barks, and roots [100]. Besides, higher phenolic and flavonoid content is also associated with better DPPH scavenging activity indicating MO leaf extracts have better antioxidant properties [100]. Nevertheless, ethanol and methanol are favoured over distilled water/aqueous extraction as these solvents yield higher phytochemical constituents with better antioxidant properties. Moreover, the geographical location and the time of sample collection affects total phenolic and flavonoid content. For instance, leaves samples collected from different districts of Tamil Nadu, Madurai and Chennai showed differing polyphenolic contents. The former showed better antioxidant properties as opposed to the latter due to presence of higher polyphenolic contents [101]. Similarly, the highest concentrations of hydrolysable (4.494 ± 0.32 mg GAE/g dry extract) and condensed polyphenols (7.149 ± 1.74 mg QE/g dry extract) were observed in MO leaflets collected from Ramos Arizpe, Coahuila compared to samples collected from the same region [102].

#### 2.3.3 Regulatory mechanism of action of *Moringa oleifera* Lam

Similar to the previously described traditional plants, numerous in vitro and in vivo studies have revealed that MO exerts antioxidant, anti-inflammatory, anti-proliferative, and anti-cancer properties by modulating different pathways involved in inflammation, oxidative stress or apoptosis **(**Table 4**)**. Parts of plant, solvent used for extraction and phytochemical composition often influence the type of immunomodulatory properties exerted by MO.

**Table 4:** Immunomodulatory action of *Moringa oleifera*. Lam.

Solvent/part of plant	Isolation of compounds/Major compound	Study Design	Immunomodulatory mechanism	Biological activities	Ref
**Aqueous extracts of MO leaves**	MOP-3	LPS-stimulated RAW264.7 murine macrophages	↑ pinocytosis in macrophages ↑ production of NO, IL-6 and TNF-α	Immunomodulation	[103]
**Aqueous extracts of MO leaves**	MOP-2	LPS-stimulated RAW264.7 murine macrophages	↑ pinocytosis in macrophages ↑ Increase production of NO, IL-6 and TNF-α	Immunomodulation	[104]
**Dried MO Leaves powder**	Z-15	LPS-stimulated RAW264.7 murine macrophages	↓ I NO production	Anti-inflammatory	[105]
**Aqueous root extract**	MRP-1	LPS-stimulated RAW264.7 murine macrophages	↓ IL-6, IL-1B, and TNF-α ↓ mRNA of iNOS and TNF-a	Anti-inflammatory	[106]
**Ethanol extract of seeds**	MIC-1	LPS-stimulated RAW264.7 murine macrophages	↑ Nrf2 expression ↓ IL-6, IL-1β, and TNF-α ↓ iNOS and TNF-a	Anti-oxidant Anti-inflammatory	[107]
**Methanol extract of seeds**	Moringa A, Glucomoringin, Vitexin	LPS-stimulated RAW264.7 murine macrophages	↓ IL-6, IL-1β, and TNF-α ↓ mRNA expression of iNOS and TNF-α	Anti-viral Immunomodulation	[108]
**Ethanol extracts of MO leaves**	NA	TNF-α/IFN-γ-induced HaCaT human keratinocytes cells	↓ TNF-α, CCL17, IL-1β, and IL-6 mRNA expression ↓ *p*-ERK1/2 and *p*-JNK expression	Anti-Inflammatory	[108]
**Ethanolic extract of MO seeds**	Moringin	Induction of EAE with a combination of tiletamine and xylazine in C57BL/6 mice	↓ Th1 related cytokine - IFN-γ ↓ IL-17 in Th17 cells ↓ TNF-α ↑ IL-10	Anti-neuroinflammation	[109]
**Ethanolic extract of MO seeds**	Moringin	Induction of EAE with a combination of tiletamine and xylazine in C57BL/6 mice	↑ Wnt1 expression ↓ GSK3β and CK2α ↓ phosphorylation of cytoplasmic β-catenin enhanced expression of β-catenin in the nucleus ↓ Fas and cleaved caspase 9 ↑Nrf2 expression ↑ PPAR-γ levels ↓ IL-1β, IL-6, and COX-2 levels ↓ GSK3β ↓ FoxP3 transcription factor and CD4 levels	Anti-inflammation Anti-oxidant Immunomodulation	[110]
**Mo leaf extract**	Gallic acid, Chlorogenic acid, Caffeic acid, Ellagic acid, *p*-coumaric acid, ferulic acid	Induction with azoxymethane, DSS- associated with colon carcinogenesis model in CD-1 mice	↓ IL-6 and TNF-a and MCP-1 cytokines ↑IL-10 production	Chemopreventive	[111]
**MO leaf extract**	glucomoringin, Glucomoringin acetate A, Glucomoringin acetate B, Glucomoringin acetate C	Human colon cancer cells - HT-29 and HCT116 Normal colon cells (CCD33Co)	↓ TNF-α and IL-1β ↑ apoptosis, Bax, Cyt *c* ↓ Bcl-2	Anti-proliferative	[112]
**Ethanolic extract of MO leaves**	isopropyl isothiocyanate, d-allose, Cetene	HCT-8 iliocecal adenocarcinoma MDA-MB-231 Breast cancer cells	↑ Induction of late apoptosis ↑ cell accumulation at G2/M phase	Immunomodulation	[113]

↓: decrease/reduced, ↑: increased/elevated.

Abbreviation – NA: not applicable, IL: interleukin, CCL17: CC chemokine ligand 17, TNF-α: tumour necrosis factor-α, Bax: Bcl-2-associated X protein, cyt *c*: cytochrome *c*, Bcl-2: B-cell lymphoma 2, Nrf2: nuclear factor erythroid 2–related factor 2, GSK3β: Glycogen synthase kinase 3 β, PPAR-γ: Peroxisome proliferators–activated receptor γ, MCP-1: Monocyte Chemoattractant Protein-1, IFN: interferon, NO: nitric oxide, iNOS: inducible nitric oxide synthase, Th1: type 1 helper T cells, ERK: extracellular signal-regulated kinase, *p*-ERK: phosphorylated ERK, JNK: c-Jun N-terminal kinase, MOP: *Moringa oleifera* polysaccharide, MRP: *Moringa oleifera* root polysaccharide, MIC: *Moringa oleifera* isothiocyanate.

**Fig. 5:**
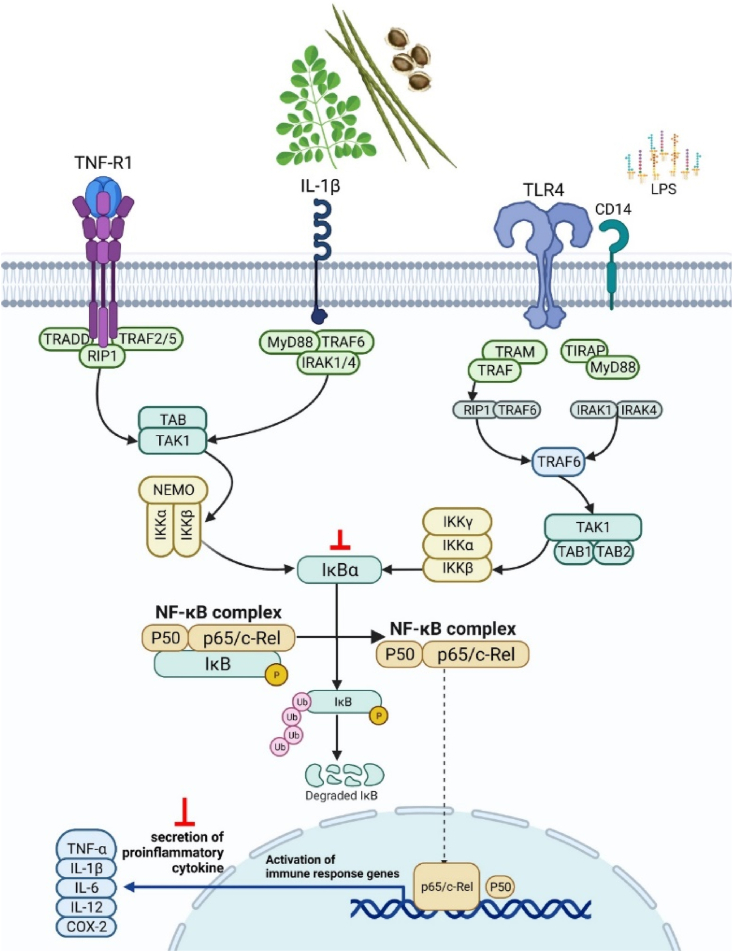
Potential immunomodulatory mechanism of action of *Moringa oleifera*. Lam Created with Biorender.com. Abbreviations – TLR: toll-like receptor, IL-1R: interleukin-1 receptor, TNFR: tumour necrosis factor receptor, MyD88: Myeloid differentiation primary response 88, IRAK: Interleukin-1 receptor-associated kinase, TIRAP: TIR domain containing adaptor protein, TRAF: tumour necrosis factor receptor–associated factor, TRAM: Toll/IL-1R domain-containing adaptor-inducing IFN-β-related adaptor molecule, TRADD: Tumour necrosis factor receptor type 1-associated DEATH domain protein, RIPK1: Receptor interacting protein 1 TAK: TGF-β-activated kinase 1, IκB: inhibitor of nuclear factor-κB, IKK: IκB kinase, NEMO: NF-kappa-B essential modulator, A20: A20 deubiquitinase, LUBAC: E3 ligase linear ubiquitin chain assembly complex, cIAP: Cellular inhibitor of apoptosis, TNF-α: tumour necrosis factor-α, COX-2: cyclooxygenase-2, IL: interleukin.

One of the main immunomodulatory mechanisms in MO is to enhance and/or activate the immune responses of macrophages through the stimulation of pattern recognition receptors (PRRs) including TLR2 and TLR4. Inflammatory responses can be induced through the binding of LPS to TLR4, which then activates cascades of pro-inflammatory mediators, transcription factors and enzymes involved in NF-κB, MAPK, and JNK signalling pathways. LPS-stimulated murine macrophages primarily increases the production of the ROS via the activation of inflammatory cytokines such as NO, IL-6, TNF-α, iNOS, COX-2, PGE_2_ and IL-1β. Hence, compounds extracted from MO modulating these cascades of pro-inflammatory mediators and inhibitors are essential as they are considered anti-inflammatory therapeutic agents (Fig. 5). For instance, expression of inflammatory mediators such as NO, iNOS, IL-1β, TNF-α and IL-6 activated by NF-κB is reduced following treatment of MO seed extract rich in isothiocyanate (MIC-1) in LPS-stimulated macrophages [107]. Interestingly, a new compound isolated from methanolic seed extract of MO, Moringa A, together with vitexin and glucomoringin strongly inhibited IL-1β, TNF-α and IL-6 triggered by H1N1 virus in RAW264.7 cells owing to its anti-viral properties [108]. Similarly, conversion of glucomoringin to moringin, and the subsequent treatment of moringin from MO seed extract suppressed neuroinflammation by suppressing expression levels of IL-6, IL-1β and COX-2 in experimental autoimmune encephalomyelitis in mouse model. Consistent with these findings, ethyl acetate fraction of MO leaves prevented the IκBα degradation and nuclear translocation of NF-κB p65 proteins, hence suppressing NF-κB pathway activation in LPS-stimulated RAW264.7 cells [114].

Furthermore, the anti-inflammatory activity of *Moringa* root polysaccharide (MRP)-1, an immunoregulatory polysaccharide isolated from MO roots consisting of rhamnose, arabinose, fructose, xylose, mannose and galactose repeating unity, was reported to suppress the NO and TNF-α production, as well as iNOS mRNA expression [106]. However, novel polysaccharides, *Moringa oleifera* polysaccharide (MOP)-2 and MOP-3, extracted from MO leaves reported otherwise as they exhibited immunomodulatory activity by stimulating gene expression and cytokine secretion of NO, IL-6 and TNF-α in LPS-stimulated RAW264.7 cells [104,103]. Unlike MRP-1, MOP-2 and MOP-3 stimulated pinocytosis in murine macrophages. It was also worth noting that MOP-3 (4.033 × 106 Da) had better immune-enhancing activity possibly due to higher molecular weight as phytochemicals with molecular weights >100 kDa were reported to possess higher immunomodulating activity [104,103].

Apart from NFKB pathway, phytochemicals from MO extracts also modulate nuclear factor erythroid 2–related factor 2 (Nrf2) pathway as it is directly or indirectly associated with inflammatory responses. Presence of bioactive compounds such as flavonoids or glucosinolates present in the leaves or seeds have been reported to be promoter of antioxidant enzymes. MIC-1 compound isolated from MO seeds were reported to bind to antioxidant response element (ARE) promoter sequences that includes regulatory proteins such as NAD(P)H Quinone Dehydrogenase 1 (NQO1), Heme oxygenase-1 (HO-1), Glutathione S-transferase Pi (GSTP1) from Nrf2 pathway [107]. MIC-1 was reported to upregulate GSTP1 caused by oxidative stress alleviating pathological events associated with acute and chronic ulcerative colitis. Furthermore, in vivo mouse model investigating the chemopreventive effect of MO on colitis-associated carcinogenesis model reported similar results as treatment with 10% and 20% MO leaves powder significantly increased enzymatic activities of NQO1 and GST in liver and colon [111]. Chemopreventive agents are reported to activate Nrf2 pathway by repressing NFKB pathway. Kelch-like ECH-associated protein 1 (Keap1) dissociates from Nrf2, binds to IKKβ, a member of the IKK complex, promoting its ubiquitination and degradation, in such a way that dissociated Keap1 plays a crucial role in the negative regulation of the NF-κB pathway. In this way, the negative regulation of IL-2, IL-6, and TNF-α and the increase of antioxidant enzymes found in this study could explain the anti-inflammatory mechanism of MO consumption by activating Nrf2/Keap1 complex and inhibiting NF-κB. Additionally, treatment of moringin isolated from MO seeds also upregulated Nrf2 gene expression indicating the potential role of MO in the treatment of autoimmune diseases such as multiple sclerosis. Moringin pretreatment also augmented antioxidant Nrf2 expression in experimental autoimmune encephalomyelitis mice [110,109]. Moringin regulated reduction of glycogen synthase kinase-3 beta (GSK3B) enhanced expression of Nrf2 expression.

MO extracts were reported to have several immunomodulatory effects in inflammation-related autoimmune diseases such multiple sclerosis [110,109], systemic lupus erythematosus [115], atopic dermatitis and rheumatoid arthritis. Onset disease progression of RA are characterised by overproduction of pro-inflammatory mediators such as IL-1, IL-6 and TNF-α. Evidently, these molecules are targeted by various MO extracts contributing to its anti-inflammatory properties. In keeping with this statement, the expression of these cytokines were reduced in complete Freund-adjuvant induced arthritis rat models of RA upon treatment with seed or leaf extract of MO [116]. Similarly, randomised human clinical trial experiments revealed that MO extracts inhibited the IL-6 production suggesting the immunosuppressive role of MO extracts in RA patients. Furthermore, experimental autoimmune encephalomyelitis, a mouse model multiple sclerosis was modulated by MO extract via the Wnt-β catenin signalling pathway by reducing the expression of GSK3B and protein kinase (Ck2)-a, inhibiting the phosphorylation of cytoplasmic B-catenin and enhancing the expression of B-catenin in the nucleus [110].

Next, MO extracts were reported to have anti-proliferative and anti-cancer effects on several cancer cell lines such breast cancer (MCF7 and MDA-MB-231), liver hepatocellular carcinoma (HepG2), lung adenocarcinoma cell (A549), ileocecal adenocarcinoma (HCT-8), colorectal carcinoma (HT-29, CaCo2 and HCT116), and leukaemia T cell (Jurkat). Potent cytotoxic effects with low IC_50_ values were observed in several cancer cells lines upon treatment with leaves and barks but not seed extracts of MO [113]. In vivo mouse model studies reported that treatment of aqueous leaf extract of MO decreased tumour volume may be contributed by bioactive compounds with anti-cancer properties such as quinic acid and palmitic acid. Apart from that, minimal toxicity were observed in healthy peripheral blood mononuclear cells and non-cancerous HEK293 cells indicating MO extracts exert anti-proliferative effects only on cancer cells but not on normal cells. Cancer cells proliferate excessively by disrupting cell cycle and apoptosis which often leads to carcinogenesis. Therefore, balance between cell proliferation and apoptosis is crucial to maintain regular cell homeostasis. The definite mechanism of action in which MO extracts inhibit cancer cells has not been fully established. In order to prevent proliferation of cancer cells, the MO extracts initiate cell cycle arrest and induces early or late apoptosis. Aqueous leaf extract of MO reduced cells in the G1, S, and G2-M phase in A549 cells while there was no significant cell cycle arrest in Hep-2 and EAC cells indicating that apoptosis may proceed with or without cell cycle arrest [113].

Furthermore, dichloromethane leaf extracts of MO were found to induce apoptosis at early stages in MCF7 cells but apoptosis at later stages were also observed due to dosage increase. Hence, the cell cycle arrest and stages when apoptosis are is induced are highly dependent on time and dosage of MO extracts. Experimental findings suggest that MO extracts also inhibited cell proliferation by reducing adenosine triphosphate (ATP) levels, increasing ROS production, eventually inducing apoptosis via mitochondria as it is an important source of ROS production [117]. Treatment of MO extracts induces both intrinsic and extrinsic apoptotic signalling pathways via mitochondria. The intrinsic apoptotic signalling pathway is activated upon sequestration of ATP to form apoptosome allowing cleavage and activation of caspase-9 protein and its downstream effector proteins. On the one hand, several experimental findings have revealed that the aqueous leaf extracts of MO primarily induced apoptosis via the intrinsic apoptotic signalling pathway by increasing the expressions of caspases (caspase-9 and caspase-3/7), cleavage of Poly (ADP-ribose) polymerase 1 (PARP-1) protein, pro-apoptotic factors (Bax, p53, Smac/DIABLO and cytochrome *c*) while reducing the expression of anti-apoptotic proteins phosphorylated B-cell lymphoma 2 (*p*-Bcl2) in A549, HepG2, and SNO [[117], [118], [119]]. On the other hand, MCF7 breast cancer cells treated with dichloromethane extract of MO leaves induced the extrinsic apoptotic signalling by significantly increasing the expression of caspase 8, p53 and Bax with no significant changes in expression levels of cytochrome *c* [120]. These results suggest that activation of intrinsic or extrinsic apoptotic signalling highly depends on the polarity of the solvents used for extractions.

## 3 Why plant immunomodulators are better than synthetic immunomodulators?

Both synthetic and natural immunomodulatory drugs are used in the clinical settings to treat various diseases. Some examples of primarily available chemotherapeutic agents used in cancer treatment include methotrexate, cisplatin, doxorubicin and cyclophosphamide. Meanwhile, non-steroidal anti-inflammatory, glucocorticoids, disease-modifying antirheumatic drugs are commonly used for the treatment of autoimmune inflammatory diseases such as RA, inflammatory bowel disease and psoriasis. Although the use of different types of synthetic immunomodulators are well established in the clinical settings, substantial toxicity and adverse side effects such as hepatoxicity, gastrointestinal toxicity and neurotoxicity were reported to be jeopardising patients’ lives. Hence, natural immunomodulators derived from traditional plants are considered as a safer substitute for chemical/synthetic drugs due to their low toxicity and less severe side effects on humans. Despite being used since ancient civilisation in traditional medicine, safety and toxicity or medicinal plants should be scientifically reported prior use in clinical settings or as a drug in public healthcare system [121]. Toxicology evaluation studies such as cytotoxicity, genotoxicity, mutagenicity, carcinogenicity, and reproductive and developmental toxicity should be included while studying the safety profile of plant-derived immunomodulatory drugs [121]. Table summarises the experimental findings of safety and toxicity assessment in *Panax ginseng* C.A. Meyer, *Curcuma longa* L. and MO.

As a first step to determining safety of an herbal drug, genotoxicity tests should be performed due to its direct correlation with cancer development. A series of battery tests involving in vitro pro- and eukaryotic systems and in vivo tests with and without metabolic systems are used in genotoxic studies [121]. Bacterial reverse mutation test (Ames test) and micronuclei test in human mammalians are examples of in vitro test for genotoxicity [121]. On the other hand, chromosomal aberration test is an example of in vivo genotoxicity test [121]. There are plenty of evidence that indicate the non-genotoxic and non-mutagenic properties of *Panax ginseng* C.A. Meyer, *Curcuma longa* L. and MO. For instance, standardized turmeric extract, namely activated curcumin C3 complex and Curcugen, were reported to show non-genotoxic and non-mutagenic potential as no apparent increase in revertant colonies were observed with or without metabolic activation in Ames test [122,123]. Increasing dosage in Ames test also showed no significant increase in revertant colonies further supporting its non-genotoxic and mutagenic properties. Similar results were obtained with increasing dosage of pectin lyase modified ginseng extract, GS-E3D ranging from 39.1 to 5000 μg/plant in bacterial reverse mutation test [124]. Furthermore, in vitro mammalian chromosomal aberration test is another common method to investigate the clastogenicity of herbal products. The primary function of this test is to evaluate numerical and structural chromosomal damage caused by phytochemicals of traditional/herbal plants. GS-E3D ginseng extract doses ranging from 313 to 2500 μg/ml while Curcugen doses ranging from 1.95 to 31.25 μg/mL showed no significant changes in structural and numerical chromosomal aberrations in Chinese Hamster lung cells and human peripheral lymphocytes, respectively [122,124]. On the other hand, in vivo micronuclei test investigating structures of chromosomal aberration in bone marrow of rodents showed no significant changes in polychromatic, micronucleated polychromatic and normochromatic erythrocytes ratio when treated with Curcugen, powdered MO leaves and MO leaves infusion and powder [122,125,126].

Pre-clinical safety evaluations assessing the acute and chronic toxicity of medicinal plants are normally carried on animal models, with strict compliance to organisation of economic cooperation and development (OECD) guidelines [127]. Acute toxicity is performed by administrating a single dose of the test drug and observing signs of mortality or toxicity for a period of 14 days to determine the half lethal dose value (LD50) of a specific chemical compound [127]. If the LD50 value of a tested compound is lower, this indicates that the compound is highly toxic. Typically, LD50 value lower than 500 mg/kg is highly toxic, ranging from 500 to 1000 mg/kg is considered moderately toxic while those values ranging from 1000 to 2000 mg/kg low in toxicity [128]. According to acute oral toxicity and LD50 values, plants extracts of *Panax ginseng* C.A. Meyer, *Curcuma longa* L. and MO were reported to be either low toxic or non-toxic [[122], [123], [124],^129^,^130^]. For example, acute toxicity and LD50 assessment of ethanolic extract of MO leaves in albino rats and rabbits were reported to be > 6616.17 mg/kg suggesting the extract is non-toxic [129]. Similarly, Curcugen was reported to be non-toxic as the acute toxicity test in Sprague-Dawley rats recorded LD50 of greater than 5000 mg/kg [122]. Another study conducted by Ohiagu and colleagues also reported LD50 value of greater than 5000 mg/kg suggesting the non-toxic behaviour of ethanolic extract of turmeric [131]. Besides, the acute toxicity study of GS-E3D ginseng extract also reported the same LD50 value as Curcugen [122,124]. Apart from assessment of LD50 value, no mortality, changes in behaviour, clinical signs and necropsy were reported in acute toxicity tests of *Panax ginseng* C.A. Meyer, *Curcuma longa* L. and MO. As for subacute and chronic toxicity evaluation, animals are subjected to repeated doses of the test compound for a time period of either 28 days, 90 days or 12 months [121,127]. The toxicity parameters that are used in evaluating sub-acute and chronic toxicity are NOAEL (no observed adverse effect level), NOEL (no observed effect level), LOAEL (lowest observed adverse effect level), and LOEL (lowest observed effect level) [127]. Treatment groups with highest dosage were reported to be non-toxic subchronic toxicity tests suggesting that turmeric, ginseng and MO extracts were non-toxic. Moreover, there were no mortality, changes in clinical, behaviour, body weight, feed consumption and adverse effects in vital organs observed during the toxicity evaluation period [122,123,[130], [131], [132], [133]].

Based on the above-mentioned studies, *Panax ginseng* C.A. Meyer, *Curcuma longa* L. and MO are considered to safe and non-toxic as they exhibit hepatoprotective, ameliorative, antioxidant, anti-fibrogenetic and anti-inflammatory properties to modulate toxicity caused by several synthetic immunomodulators or environmental toxins. However, further toxicological studies are needed for standardized plant extracts to confirm its safety and efficacy in pre-clinical investigations before being used clinical studies.

## 4 Limitations and challenges from plant-derived immunomodulators

**Fig. 6:**
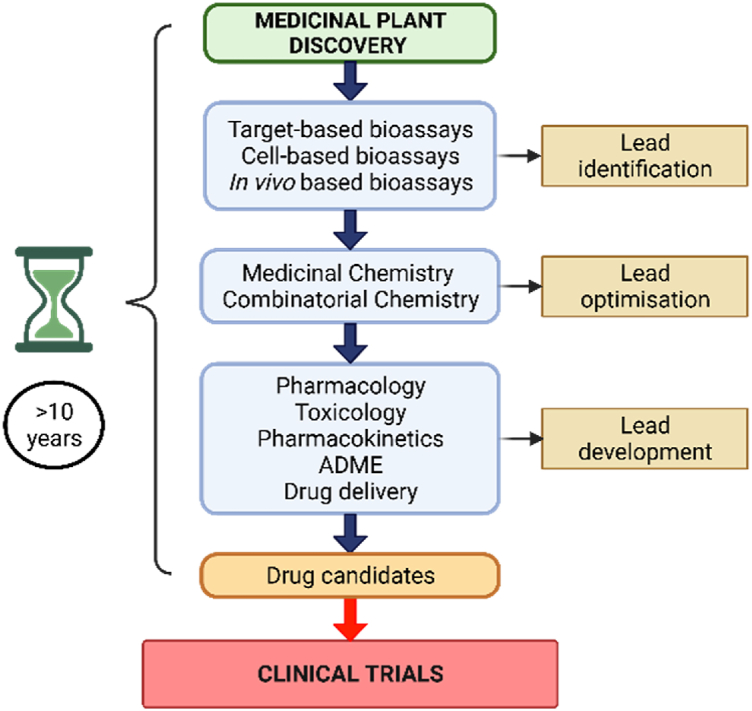
Schematic of typical medicinal plant drug discovery and development. Adapted from Balunas and Kinghorn, 2005.

Despite numerous health benefits, researchers face challenges and limitations in discovering and preparing immunomodulatory drugs derived from traditional plants. Selecting and identifying plant material, harvesting, isolation, characterization, implementing appropriate screening bioassays and synthesis, testing their efficacy and toxicity and performing clinical trials are some challenges faced in successfully developing plant-derived compounds [134]. Moreover, the lengthy process of drug discovery from plant or natural that includes lead optimization, lead development and clinical trials is time-consuming and expensive (Fig. 6) [135]. Another limitation of immunomodulatory drugs derived from natural, or plant sources is the poor bioavailability of isolated compounds [136]. Problems such as failure to maintain activity after extraction, isolation process leading to mundane compounds, and a low fraction of final product for structure elucidation often result in poor bioavailability of phytocompounds [137]. Intrinsic toxicity of medicinal plants is highly associated with the presence of pharmacologically active constituents [138].

## 5 Concluding remarks

Various traditional plants have been used for treating and managing illnesses and immunological disorders for ages. It is evident that *Curcuma longa*. L, *Panax ginseng* C. A. Meyer., and *Moringa oleifera*. Lam stimulates, suppresses, and enhances the body's innate and adaptive immune systems to maintain homeostasis and defend against pathogens. These traditional plants exert antioxidant, anti-inflammatory, anti-proliferative, anti-cancer and immunomodulatory properties by inhibiting various pathways that are involved in inflammation and apoptosis such as NF-κB, MAPK, intrinsic and extrinsic mitochondrial apoptotic pathway. In general, these pathways produce pro-inflammatory cytokines such as IL-1β, IL-6 and TNF-α that are primarily inhibited by plant immunomodulators. Due to the adverse side effect of currently available chemically synthesized drugs in the market, such as cyclosporine, cyclophosphamide, and azathioprine, there is an urgent need to identify immunomodulatory agents from plants to be used as stand-alone drugs or in combinatorial immunotherapy to treat immunological disorder, especially cancer and autoimmune diseases. Even though phytocompounds derived from plants demonstrate potential immunomodulatory properties, challenges faced during extraction and purification result in poor bioavailability limits and slow down the process of unravelling the full potential of immunomodulatory compounds. Researchers have now developed and adapted nanotechnology-based formulations and alterations in drug administration techniques to reduce these disadvantages. Although phytocompounds from *Curcuma longa*. L, *Panax ginseng* C. A. Meyer., and *Moringa oleifera*. Lam has demonstrated a great potential to influence the immune system in vitro and in vivo; the lack of clinical trial data to verify its efficacy on people justifies the demand for a more systematic and operationally thorough experiment involving standardized plant extracts backed adequately by pharmacokinetics toxicology reports.

## Funding

This study was supported by the Universiti Sains Malaysia (Short-term Research Grant) No.304/PTEKIND/6315523 granted to Ana Masara Ahmad Mokhtar.

## CRediT authorship contribution statement

**Muggunna Balasubramaniam:** Writing – review & editing, Writing – original draft, Data curation, Conceptualization. **Sarah Sapuan:** Supervision. **Ilie Fadzilah Hashim:** Supervision, Funding acquisition. **Nurul Izza Ismail:** Funding acquisition, Supervision. **Amira Suriaty Yaakop:** Funding acquisition, Supervision. **Nur Azzalia Kamaruzaman:** Funding acquisition, Supervision. **Ana Masara Ahmad Mokhtar:** Writing – review & editing, Validation, Supervision, Funding acquisition.

## Declaration of competing interest

The authors declare that they have no known competing financial interests or personal relationships that could have appeared to influence the work reported in this paper.
